# Thermodynamic Entropy as a Noether Invariant from Contact Geometry

**DOI:** 10.3390/e25071082

**Published:** 2023-07-19

**Authors:** Alessandro Bravetti, Miguel Ángel García-Ariza, Diego Tapias

**Affiliations:** 1 Instituto de Investigaciones en Matemáticas Aplicadas y en Sistemas, Universidad Nacional Autónoma de Mexico, A. P. 70543, Ciudad de Mexico 04510, Mexico; miguel.garcia@iimas.unam.mx; 2 Institute for Theoretical Physics, University of Göttingen, 37073 Göttingen, Germany; diego.tapias@theorie.physik.uni-goettingen.de

**Keywords:** Noether’s theorem, Hamiltonian systems, contact geometry, entropy, thermostatted systems

## Abstract

We use a formulation of Noether’s theorem for contact Hamiltonian systems to derive a relation between the thermodynamic entropy and the Noether invariant associated with time-translational symmetry. In the particular case of thermostatted systems at equilibrium, we show that the total entropy of the system plus the reservoir are conserved as a consequence thereof. Our results contribute to understanding thermodynamic entropy from a geometric point of view.

## 1. Introduction

Geometric methods have become indispensable in modern theoretical physics. A primary example is Noether’s theorem, which in its most famous version, relates the symmetries of a mechanical system to the corresponding conservation laws. In thermodynamics, Noether’s theorem has not had thus far the same success as, for instance, in mechanics. This is certainly to be ascribed to the fact that, despite many attempts, there is still no consensus on a variational or geometric formulation of thermodynamics, especially in the irreversible case (see, e.g., [1,2,3,4,5,6] and references therein). Nevertheless, a lot of thermodynamics is about symmetries and conserved quantities. Therefore, it would be very insightful to extend Noether’s analysis to this context. Two recent attempts in this direction are particularly interesting for us.

The first one is motivated by the seminal work of Wald [7], proving that the entropy of a black hole is the Noether invariant associated with the horizon Killing field. A crucial question naturally arising is whether entropy is also a Noether invariant for standard systems. Recently, Sasa and Yokokura proved that for thermally isolated systems (systems enclosed by adiabatic walls) under the action of an external control parameter this is indeed the case [8]. To do so, they used the standard action principle of mechanics in conjunction with a generalized version of Noether’s theorem, which applies to (dynamical) symmetries that do not necessarily leave the action invariant [9].

A different approach to the use of the standard Noether theorem in statistical mechanics was considered in references [10,11]. There, the key idea was to exploit the invariance of global thermodynamic potentials under symmetries of the mechanical Hamiltonian to derive new identities, the so-called *Noether identities*, for systems in different ensembles.

Motivated by the above, we use Noether’s theorem to derive a general identity for the Gibbs’ entropy associated with the stationary (but not necessarily equilibrium) state of a contact Hamiltonian system. Specifically, we show that this entropy can be seen as the average of the Noether invariant associated with the symmetry under time translations. In the particular case of systems in equilibrium with a heat bath, we show that the identity becomes a conservation law for the total entropy of the system plus the reservoir. To this end, we rely on recent results showing that a large class of systems that do not preserve energy can be modelled through contact Hamiltonian dynamics [12,13,14], which admits a variational formulation through Herglotz’s variational principle [15,16,17]. This framework yields a geometric characterization and generalization of the classical Nosé–Hoover equations [18,19,20,21], providing a microscopic dynamics consistent with the physical situation of a mechanical system in thermal equilibrium with a heat bath [22].

To carry out what we described above, we have organized this paper as follows: We present a summary of ref. [8] in Section 2 that allows for a clear distinction between the results presented therein and our own work. We dedicate Section 3 to introducing contact dynamics from the Hamiltonian approach, which we use to model the dynamics of non-conservative systems. We then proceed in Section 4 to show how Noether’s theorem implies an identity for Gibbs’ entropy of any (possibly non-equilibrium) stationary state in this framework. In Section 5, we apply this result to systems in thermal equilibrium with a heat bath, showing that the conservation of the total entropy is a consequence of the time-translational symmetry. Finally, we present our concluding remarks and perspectives for future work in Section 6.

## 2. Entropy as a Noether Invariant from the Standard Variational Principle

Entropy appears as a Noether invariant when considering thermally isolated systems in the quasistatic regime. This was proven by Sasa and Yokokura from a variational perspective [8]. In this section, we provide a brief summary of their derivation in order to contrast it with our own result.

Consider a system of *N* particles interacting only through conservative forces. Such a system can be described by a Lagrangian function $L(q,\dot{q},\alpha )$, with corresponding action

(1) $$A\left(\hat{q},\hat{\alpha }\right):=\int_{t_{i}}^{t_{f}}L\left(q,\dot{q},\alpha \right)dt\;,$$

where $\hat{q}=q\left(t\right)$, with $t\in [t_{i},t_{f}]$, is the trajectory, and α is an extensive control parameter (typically the volume), which is changed according to a prescribed protocol $\hat{\alpha }=\alpha \left(t\right)$.

To prove that entropy is a Noether invariant, the authors of ref. [8] start with a generalized version of Noether’s theorem, first proved in ref. [9]. Let us recall this theorem in the language of the former.

**Theorem** **1** (Generalized Noether)**.** *Let $\bar{t}=t+\eta \xi \left(q,\dot{q},\alpha \right)$ be a one-parameter group of non-uniform time translations, where η is a small parameter. Suppose that for some $\hat{\alpha }$ there exist $\xi (q,\dot{q},\alpha )$ and $\psi (q,\dot{q},\alpha )$ such that the corresponding variation of the action is*

(2) $$\delta A=\eta \int_{t_{i}}^{t_{f}}\frac{d\psi }{dt}dt\;.$$

*Then, the transformation $\bar{t}$ constitutes a dynamical symmetry, with a corresponding constant of motion*

(3) N:=ψ+Eξ,

*where $E={\dot{q}}_{i}\frac{\partial L}{\partial {\dot{q}}_{i}}-L$ is the energy of the system.*

More concretely, ref. [8] considers that

(4) $$\bar{t}=t+\eta \hbar \beta \;,$$

with β the inverse temperature (Boltzmann’s constant is set to unity). Then, the use of Theorem 1 plus some physical reasoning leads to the quantity

(5) N=ℏS+bℏN

as the corresponding constant of motion, with *S* being the thermodynamic entropy of the system and *b* a dimensionless constant. As the number of particles *N* is fixed, this entails that the entropy is the conserved quantity associated with the symmetry (2).

As emphasized in ref. [8], the theorem applies in general to systems that are thermally isolated (this means adiabatic in thermodynamics language). In the particular case of an ideally fully isolated system, the adiabatic theorem ensures that the entropy takes a constant value along almost all trajectories in the quasistatic limit. Thus, it seems natural to have the entropy as the Noether invariant. The situation is more involved in the generic thermally isolated case, as the trajectories of the system are not necessarily solutions of the Lagrangian equations of motion. Nevertheless, if one restricts to the so-called *thermodynamically consistent trajectories*, that is, those curves for which the mechanical work coincides in the quasistatic limit with the thermodynamic work, then the entropy is still the conserved quantity corresponding to the symmetry (2).

It is remarkable that, in this framework, the characterization of the entropy as the unique Noether invariant depends explicitly on the consideration of quasistatic processes with a time-dependent control parameter, and on the restriction to thermodynamically consistent trajectories. Our main result, in contrast, considers systems at equilibrium not subject to an external protocol, and it does not need to restrict to a special class of trajectories. Indeed, almost all the trajectories in our case are thermodynamically consistent (see Section 5).

## 3. Contact Hamiltonian Dynamics

In this section, we give a concise introduction to contact dynamics in its Hamiltonian formulations. The aim of this section is to introduce the results that will be relevant in the following. We point to refs. [12,13,14] for a deeper discussion on contact Hamiltonian systems and their applications and to [15,16] for their Lagrangian formulation (see also [23,24]).

Contact Hamiltonian dynamics is the analogue to the contact manifolds of standard Hamiltonian dynamics on symplectic manifolds [12,13,14].

*A contact manifold* is defined as a (2n+1)-dimensional manifold endowed with a 1-form η satisfying $\eta \wedge {\left(d\eta \right)}^{n}\neq 0$, where ∧ is the wedge product and d is the exterior differential. More precisely, this corresponds to an *exact* or *co-oriented* contact manifold (see, e.g., [25,26]). The latter is endowed with a unique vector field R, called *Reeb vector field*, satisfying $\iota_{R}d\eta =0$ and $\iota_{R}\eta =1$, where ι stands for the inner contraction of vectors with forms.

The dynamics of a contact manifold is usually given in terms of a *contact Hamiltonian vector field* $X_{H}$, defined as the only vector field satisfying

(6) $$\iota_{X_{H}}d\eta =dH-R\left(H\right)\eta \;\mathrm{and}\;\iota_{X_{H}}\eta =-H\;,$$

where H is a differentiable function on the manifold called the *contact Hamiltonian*.

Locally, on any contact manifold there are “canonical” (Darboux) coordinates $\left(q^{i},p_{i},s\right)$ such that the local expressions of the contact 1-form and the Reeb vector field are $\eta =ds-p_{i}dq^{i}$ and R=∂/∂s, respectively. The dynamical equations corresponding to the equations for the integral curves of $X_{H}$ read

(7) $$\begin{array}{c} {\dot{q}}^{i}=\frac{\partial H}{\partial p_{i}},\;\dot{p_{i}}=-\frac{\partial H}{\partial q^{i}}-p_{i}\frac{\partial H}{\partial s},\;\dot{s}=p_{i}\frac{\partial H}{\partial p_{i}}-H\;. \end{array}$$

The main characteristic of these systems is the fact that, when the Hamiltonian does not depend explicitly on *s*, all the results of standard symplectic Hamiltonian dynamics such as conservation of the Hamiltonian and Liouville’s theorem are recovered. On the other hand, when H does depend on *s*, the Hamiltonian is not conserved and the evolution $X_{H}$ has a non-zero divergence, according to the general expressions

(8) $$\dot{H}=X_{H}\left(H\right)=-H\frac{\partial H}{\partial s}\;$$

and

(9) $$\mathrm{div}\left(X_{H}\right)=-\left(n+1\right)\frac{\partial H}{\partial s}\;,$$

where *n* is the number of degrees of freedom of the system, and the divergence of a vector field *Y*, div(Y), is defined as the unique real-valued function satisfying ${£}_{Y}\left(\eta \wedge {\left(d\eta \right)}^{n}\right)=\mathrm{div}Y\;\left(\eta \wedge {\left(d\eta \right)}^{n}\right)\;,$ where *£* denotes the Lie derivative.

## 4. A Noether Identity from Contact Geometry

Starting from the results of the previous section, one can prove a contact version of Noether’s theorem [13,27,28,29], which we now recall (see also [15,16] for the Lagrangian counterpart and further discussion).

We begin with the following definition.

**Definition** **2.** *An infinitesimal dynamical symmetry of a contact Hamiltonian system $X_{H}$ is a vector field Y∈X(M) such that $[Y,X_{H}]=0$.*

Now, by analogy with the standard Noether theorem, we can state the following relation between infinitesimal dynamical symmetries and conserved quantities in the contact case.

**Theorem** **3** (Contact Noether)**.** *Let Y be an infinitesimal dynamical symmetry of the contact Hamiltonian system $X_{H}$. Then, the quantity*

(10) $$\exp\left(\int_{0}^{t}\frac{\partial H}{\partial s}d\theta \right)\iota_{Y}\eta$$

*is conserved along the solutions of (7).*

**Proof.**  Let $N:=\exp\left(\int_{0}^{t}\partial H/\partial sd\theta \right)\iota_{Y}\eta$. Then,

(11) $$\begin{array}{cc} X_{H}\left(N\right) & =\exp\left(\int_{0}^{t}\frac{\partial H}{\partial s}d\theta \right)\left[\frac{\partial H}{\partial s}\iota_{Y}\eta +{£}_{X_{H}}\left(\iota_{Y}\eta \right)\right]\\ \; & =\exp\left(\int_{0}^{t}\frac{\partial H}{\partial s}d\theta \right)\left[\frac{\partial H}{\partial s}\iota_{Y}\eta +\iota_{\left[X_{H},Y\right]}\eta +\iota_{Y}{£}_{X_{H}}\eta \right]. \end{array}$$

On the other hand, substituting Equation (6) in Cartan’s formula, ${£}_{X_{H}}\eta =\iota_{X_{H}}d\eta +d\iota_{X_{H}}\eta$, yields

(12) $${£}_{X_{H}}\eta =-R\left(H\right)\eta .$$

Finally, since R(H)=∂H/∂s and $[Y,X_{H}]=0$, the result follows. □

It is worth observing that, typically, the quantity (10) is the product of the classical Noether invariant with the exponential factor $\exp\left(\int_{0}^{t}\frac{\partial H}{\partial s}d\theta \right)$.

For instance, when H does not depend explicitly on *t*, the system is invariant under time translations and the corresponding generator is just $Y=X_{H}$. Therefore, using the second condition in (6), the associated Noether invariant reads (up to a sign)

(13) $$E:=\exp\left(\int_{0}^{t}\frac{\partial H}{\partial s}d\theta \right)H\;,$$

which is the analogue of the standard conservation of the energy. Indeed, if H does not depend on *s*, then the conservation of (13) precisely reduces to the conservation of the energy.

Our purpose is to apply Theorem 3 to systems in the specific states we introduce below.

**Definition** **4.** *Let $X_{H}$ be a contact Hamiltonian system. We say that $X_{H}$ is in a stationary state ρ if*

$${£}_{X_{H}}\left(\rho \;\eta \wedge {\left(d\eta \right)}^{n}\right)=0\;,$$

*where £ is the Lie derivative along the flow of $X_{H}$, ρ=ρ(q,p,s) is a probability distribution and η is the contact 1-form defining the geometry of the system.*

We can now state and prove the following remarkable consequence of Theorem 3.

**Corollary** **5.** *Let $X_{H}$ be a contact Hamiltonian system in a stationary state ρ that depends only on H, with H being a positive function. Then, the quantity*

(14) $$I=-\ln\rho -\int_{0}^{t}\mathrm{div}\left(X_{H}\right)d\theta \;,$$

*is the Noether invariant associated with the time-translational symmetry.*

**Proof.**  We begin by noticing that, by (13), the Noether invariant associated with the symmetry under time translations can be written as

(15) $$\ln\left(E\right)=\ln H+\int_{0}^{t}\frac{\partial H}{\partial s}d\theta \;.$$

Let us assume that the system is completely described by a positive Hamiltonian function H (cf. Equation (21) below). Then, ρ=ρ(H) and, from Liouville’s theorem for contact Hamiltonian systems [30], it follows that

(16) $$\rho =\frac{H^{-(n+1)}}{Z}\;,$$

where Z is a normalization constant, is the unique stationary state for which ρ is a function of H only [31] (see ref. [32] for the general case).

Thus, using (9) and (16) (and multiplicating by n+1), we can rewrite the Noether invariant (15) as in (14). □

Note that the invariant I has been introduced by Fukuda and Nakamura [33] with multiple applications, especially in the field of molecular dynamics [34,35]. Here, however, we have proved that this quantity is invariant for any general time-independent contact system, and that it is exactly the Noether invariant associated with the symmetry under time translations.

Let us consider now Gibbs’ entropy for the state ρ,

(17) $$S\left(\rho \right):=-\int_{\Gamma }\rho \;\ln\rho \;dq\;dp\;ds\;,$$

where Γ is the corresponding contact manifold.

The conservation of the Noether invariant I in Corollary 5 implies the following *Noether identity for Gibbs’ entropy*:

(18) $$S\left(\rho \left(t\right)\right)=S\left(\rho \left(0\right)\right)+S_{\mathrm{irr}}\left(t\right)\;\forall t\geq 0\;,$$

where $S_{\mathrm{irr}}\left(t\right):=\int_{0}^{t}\langle \mathrm{div}\left(X_{H}\right)\rangle d\theta$ is the total irreversible entropy production.

Indeed, a standard calculation (see, e.g., refs. [36,37]) shows that, for any (possibly non-equilibrium) stationary state ρ, the time derivative of *S* along the flow of a vector field $X_{H}$ is

(19) $$\begin{array}{c} \dot{S}=\langle \mathrm{div}\left(X_{H}\right)\rangle \;. \end{array}$$

Moreover, by averaging (14) with ρ, we obtain the sum of two terms, the first of which is Gibbs’ entropy of ρ at time *t*, and the second one is, using (19), the total entropy production up to time *t*. We conclude that

(20) $$S\left(\rho \left(0\right)\right)\equiv \langle I\rangle =S\left(\rho \left(t\right)\right)-S_{\mathrm{irr}}\left(t\right)\;,$$

which, upon replacing, is the above identity (18).

Summing up, we have shown so far that for general contact systems the Noether invariant associated with time-translational invariance is given by (14), and that its conservation implies an identity for Gibbs’ entropy of any (possibly non-equilibrium) stationary state. Equation (18) can be seen as a *Noether identity*, following the language of references [10,11]. We remark, however, that so far this identity is a general relation for the Gibbs’ entropy of any stationary state that depends only on H. In the next section, we will consider an important special case of this identity, one in which a connection with the thermodynamic entropy is possible.

## 5. Entropy as a Noether Invariant for Systems at Thermal Equilibrium

In this section, we consider thermostatted mechanical systems in the context of contact dynamics and show that the results of the previous section imply that the total entropy of the system plus the reservoir is conserved.

Given a mechanical system with *n* degrees of freedom described by a Hamiltonian $H_{\mathrm{mec}}\left(q,p\right)$, which is in thermal equilibrium with a reservoir, its dynamics can be described on the contact manifold $\Gamma =T^{*}Q\times R$, where $T^{*}Q$ is the standard phase space (cotangent bundle) of the system, endowed with the corresponding contact Hamiltonian [12,22,34]

(21) $$H_{\mathrm{eq}}={\left(\rho_{\mathrm{th}}\left(q,p\right)f\left(s\right)\right)}^{-1/(n+1)}\;,$$

where $\rho_{\mathrm{th}}$ is the thermal equilibrium distribution, i.e.,

$$\rho_{\mathrm{th}}=\exp\left(-\beta H_{\mathrm{mec}}\right)/Z_{\mathrm{th}},$$

and f(s) is a (free-to-choose) distribution for the variable *s* that controls the temperature (for instance, by choosing f(s) to be a Gaussian distribution, the dynamics generated by (21) leads to the well-known Nosé–Hoover equations [38]). Assuming ergodicity, for these types of dynamics almost all the trajectories of the system are consistent with thermodynamics [22], i.e., they are thermodynamically consistent.

An important observation is that for such systems, from (17) and (21), we have

(22) $$S\left(\rho \left(t\right)\right)=S\left(\rho_{\mathrm{th}}\right)+S\left(f\left(s\right)\right)=S_{\mathrm{sys}}+S_{\mathrm{res}}\;,$$

that is, S(ρ(t)) is the total thermodynamic entropy of the system plus the reservoir.

Moreover, since $H_{\mathrm{eq}}$ is time-independent, we have a symmetry under time translations, and the associated Noether invariant is given by (14). This implies that identity (18) holds, yielding the following remarkable result.

**Theorem** **6** (Main)**.** *For a system of n degrees of freedom at thermal equilibrium with a reservoir—a system described by (21)—the conservation of the Noether invariant I implies the conservation of the thermodynamic entropy of the mechanical system plus the entropy of the reservoir.*

**Proof.**  From (9), we can write (19) in general as

(23) $$\dot{S}=-\left(n+1\right)\int_{\Gamma }\rho \;\frac{\partial H}{\partial s}d\Gamma \;,\;\;d\Gamma =dq\;dp\;ds\;.$$

For H as in (21), using (23), we obtain

(24) $$\begin{array}{cc} \dot{S} & =\int_{\Gamma }\rho H_{\mathrm{eq}}\frac{f^{\prime }\left(s\right)}{f(s)}d\Gamma \\ \; & =\int_{\Gamma }{\left(\rho_{\mathrm{th}}\left(q,p\right)f\left(s\right)\right)}^{n/(n+1)}\frac{f^{\prime }\left(s\right)}{f(s)}d\Gamma \\ \; & =\int_{T^{*}Q}\rho_{\mathrm{th}}^{n/(n+1)}dp\;dq\int_{R}f{\left(s\right)}^{-\frac{1}{n+1}}f^{\prime }\left(s\right)ds\\ \; & ={\left.C\;{\left(f\left(s\right)\right)}^{\frac{n}{n+1}}\right|}_{-\infty }^{\infty }=0\;, \end{array}$$

and the last equality stems from the fact that f(s) vanishes at infinity. Thus, using the identity (18), which follows from the conservation of the Noether invariant I, we obtain

(25) S(ρ(t))=S(ρ(0))∀t≥0.

This means that the total entropy of the system plus the reservoir is conserved (cf. (22)). □

The fact that the total entropy above is conserved at equilibrium is trivial. What we consider important is that, in light of Noether’s theorem, this can be regarded as a consequence of a symmetry under time translations. Indeed, we could rephrase Theorem 6 informally in the following way: *For a system described by (21), thermal equilibrium emerges as a consequence of time-translational invariance.* Moreover, since the system and the heat bath are allowed to exchange energy, the entropy of the former alone is not constant (there is a heat flow from and into it).

## 6. Conclusions

Using an appropriate geometric description for the microscopic dynamics of mechanical systems in thermal equilibrium with a heat bath, we have derived a relationship between Noether’s theorem and the conservation of the total entropy of the system plus the reservoir. We stress that in this case the symmetry related to the conservation of the entropy is just the standard time-translational invariance. Therefore, we conclude that thermodynamic equilibrium emerges as a consequence of time-translational invariance.

Our results are in line with recent findings deriving the thermodynamic entropy as the Noether invariant associated with a symmetry under (non-uniform) time translations. Indeed, it should be noted that in our setting the temperature of the system is constant (we simulate the canonical ensemble). Therefore, the non-uniform time translation of Equation (4) found in [8] consistently reduces to our uniform result.

Our work also provides an answer in a deterministic setting to the question formulated in ref. [39], concerning the extension of the relationship between entropy and the Noether invariant to systems in contact with a heat bath. Despite being deterministic, the description we present here suggests viewing the Langevin equations governing the stochastic processes in those systems through the lens of stochastic contact Hamiltonian systems and the related stochastic Herglotz variational principle [40,41]. This is left for future work.

## Data Availability

Not applicable.
